# Analysis of Cerebral Spinal Fluid Drainage and Intracranial Pressure Peaks in Patients with Subarachnoid Hemorrhage

**DOI:** 10.1007/s12028-024-01981-9

**Published:** 2024-04-15

**Authors:** Anton Früh, Peter Truckenmüller, David Wasilewski, Peter Vajkoczy, Stefan Wolf, for the Earlydrain Study Group

**Affiliations:** 1 Department of Neurosurgery, Charité Universitätsmedizin Berlin, Corporate Member of Freie Universität Berlin, Humboldt-Universität zu Berlin and Berlin Institute of Health, Charitéplatz 1, 10117 Berlin, Germany; 2 BIH Charité Junior Digital Clinician Scientist Program, BIH Biomedical Innovation Academy, Charitéplatz 1, 10117 Berlin, Germany

**Keywords:** Subarachnoid hemorrhage, Cerebrospinal fluid, Lumbar drainage, Intracranial pressure

## Abstract

**Background:**

After aneurysmal subarachnoid hemorrhage (aSAH), elevated intracranial pressure (ICP) due to disrupted cerebrospinal fluid (CSF) dynamics is a critical concern. An external ventricular drainage (EVD) is commonly employed for management; however, optimal strategies remain debated. The randomized controlled Earlydrain trial showed that an additional prophylactic lumbar drainage (LD) after aneurysm treatment improves neurological outcome. We performed a post hoc investigation on the impact of drainage volumes and critical ICP values on patient outcomes after aSAH.

**Methods:**

Using raw patient data from Earlydrain, we analyzed CSF drainage amounts and ICP measurements in the first 8 days after aSAH. Outcomes were the occurrence of secondary infarctions and the score on the modified Rankin scale after 6 months, dichotomized in values of 0–2 as favorable and 3–6 as unfavorable. Repeated measurements were considered with generalized estimation equations.

**Results:**

Earlydrain recruited 287 patients, of whom 221 received an EVD and 140 received an LD. Higher EVD volumes showed a trend to more secondary infarctions (*p* = 0.09), whereas higher LD volumes were associated with less secondary infarctions (*p* = 0.009). The mean total CSF drainage was 1052 ± 659 mL and did not differ concerning infarction and neurological outcome. Maximum ICP values were higher in patients with poor outcomes but not related to drainage volumes via EVD. After adjustment for aSAH severity and total CSF drainage, higher LD volume was linked to favorable outcome (per 100 mL: odds ratio 0.61 (95% confidence interval 0.39–0.95), *p* = 0.03), whereas higher EVD amounts were associated with unfavorable outcome (per 100 mL: odds ratio 1.63 (95% confidence interval 1.05–2.54), *p* = 0.03).

**Conclusions:**

Findings indicate that effects of CSF drainage via EVD and LD differ. Higher amounts and higher proportions of LD volumes were associated with better outcomes, suggesting a potential quantity-dependent protective effect. Optimizing LD volume and mitigating ICP spikes may be a strategy to improve patient outcomes after aSAH.

*Clinical trial registration*: ClinicalTrials.gov identifier: NCT01258257.

**Supplementary Information:**

The online version contains supplementary material available at 10.1007/s12028-024-01981-9.

## Introduction

Subarachnoid hemorrhage (SAH) caused by a rupture of an intracranial aneurysm (aSAH) is a severe life-threatening emergency that is characterized by an acute onset of symptoms and is associated with considerable rates of morbidity and mortality [1–4]. The incidence is estimated at around nine cases per 100,000 person-years, displaying significant regional variations. Notably, women are more affected than men [2, 5].

The current standard of care for ruptured aneurysms, typically involving surgical clipping or endovascular coiling, is generally performed within the first 24–48 h after onset [6]. A critical manifestation of aSAH is elevated intracranial pressure (ICP), primarily due to disrupted cerebrospinal fluid (CSF) dynamics [7]. Alongside conservative methods, CSF drainage through an external ventricular drain (EVD) is commonly employed in neurocritical care units to manage elevated ICP [8, 9]. Despite its widespread use, the optimal strategy for CSF drainage and ICP management in SAH cases continues to be a topic of debate [10, 11]. A common complication of aSAH is vasospasm, which affects approximately 70% of patients after the initial bleeding event. This phenomenon complicates medical management and, in conjunction with other pathomechanisms, contributes to secondary cerebral infarctions, which are observed in approximately 40% of patients with aSAH [3, 12]. Particularly in relation to CSF drainage rates, the available data are notably limited [13, 14]. Della Pepa et al. [15] reported a beneficial effect of CSF drainage via EVD on the occurrence of vasospasm, whereas Maeda et al. [16] observed a higher incidence of secondary infarctions associated with the use of EVDs. However, neither work adjusted for the severity of the initial hemorrhage.

The randomized controlled multicenter Earlydrain trial [17] demonstrated an improvement of neurological outcome in patients with aSAH through prophylactic lumbar drainage (LD) after the aneurysm treatment. Using clinical data recorded in Earlydrain in the first 8 days after hemorrhage, we analyzed the impact of the amount of CSF drainage and ICP peaks on neurologic outcomes. The present analysis aims to analyze the actual recorded ICP data and drainage volumes of this trial, independent of the assigned randomization group. We hypothesized that LD and EVD CSF drainage volumes, as well as ICP, have an impact on the outcome of patients with aSAH.

## Methods

### Study Design and Patients

This study represents a substudy of a prospectively performed multicenter randomized clinical trial encompassing 19 centers across Germany, Switzerland, and Canada [17]. The lead ethical approval was granted by the Ethics Committee of the University of Erlangen, Germany. Additionally, each participating center received local approval from the corresponding ethics board, and the study was conducted according to the Declaration of Helsinki. This trial was registered at ClinicalTrials.gov (identifier: NCT01258257). From 2011 to 2016, adult patients with aSAH were enrolled and randomized 1:1 to receive either standard of care or standard of care plus an additional LD. The main study examined whether controlled LD has an impact on the outcome after SAH. The present work is a secondary analysis that further investigates the influence of drainage volumes and ICP. For this purpose, we conducted analyses of the primary raw data of the main study, available at the Mendeley repository [18].

### Data Collection

The study data were received in CSV file format and imported into R environments, SPSS, and GraphPad using standard import functions. Patients were treated as previously described in the published main study [17] and according to established guidelines [19, 20]. Briefly, after standard of care at the emergency departments and consecutive aneurysm treatment, the patients were randomized between LD and standard of care groups. ICP monitoring was performed according to the local standard. Patients in the LD group received an additional LD after the treatment of the aneurysm. Drainage was started when a postinterventional computed tomography (CT) scan indicated safety. A lumbar CSF drainage rate of 5 mL/h was targeted independent of ICP. Pressure transducers of EVD and LD were leveled at the same level at the external meatus. According to protocol, CSF drainage through the LD was suggested to be performed only when the difference between both drains (EVD and LD) was < 5 mm Hg and the ICP levels were < 20 mm Hg. The EVD regimen was left to the discretion of the local clinicians. In situations requiring CSF drainage, this was suggested to be performed via EVD. All drainage amounts in the first 8 days after aSAH were recorded. The total amount of CSF was defined as the cumulative volume drained within the initial 8 days following aSAH of each patient. For patients with both an EVD and LD, the total amount of CSF was calculated as the sum of the drained volumes from both EVD and LD. In cases in which only an EVD or LD was in place, the total CSF amount was equivalent to the EVD or LD drainage volume, respectively. In patients in whom the CSF volume was drained through both EVD and LD systems, an LD percentage was calculated. This was defined as the percentage of the CSF volume drained through the LD of the total drainage volume (EVD and LD) during the initial 8 days following aSAH. ICP was monitored constantly as long it was deemed appropriate to local investigators. The ICP at 7 a.m. and the highest daily value were prospectively documented for the first week. The present analysis was performed as actual treatment happened (as-treated analysis).

### Outcome Assessment

Follow-up time of the patients was until 6 months after randomization or until death. The neurological outcome was assessed using the modified Rankin scale (mRS) [21] at 6 months after the bleeding event. It was dichotomized into favorable outcome (mRS ≤ 2) and unfavorable neurological outcome (mRS > 2). Further, the occurrence of new infarctions, which had not been present in the postinterventional CT scan performed after the aneurysm treatment, was determined. These secondary infarctions were diagnosed with the last cerebral imaging (either CT or magnetic resonance imaging) before discharge from acute care. The evaluating radiologists were blinded for study events.

### Statistical Analysis

Discrete data were presented as count and percentage and analyzed by using the *χ*^2^ test. Continuous data were presented as mean and standard deviation or median and interquartile range (IQR) and compared by using the *t*-test or Mann–Whitney *U*-test. Area under the curve (AUC) was determined using receiver operating characteristic (ROC) analyses. The total amount of CSF and the percentage were analyzed and presented with the stacked bar plot function of RStudio and GraphPad Prism. ICP data and daily drainage volumes were analyzed with generalized additive models. For univariate and multivariate analysis of the rate of infarctions and the dichotomized mRS, generalized estimation equations were used. Models were adjusted according to known predictors for severity of the SAH based on the Earlydrain study [17], namely a Hunt and Hess score > 3, age, and the presence of parenchymal and intraventricular hemorrhages; comparisons between models were performed with the quasi-likelihood criterion QIC [22]. Odds ratio plots were created using the Forestploter R package from GitHub. Two-sided *p* values of less than 0.05 were used to indicate statistical significance. SPSS (version 25.0; IBM Corp., Armonk, NY), R (version 4.3.1), RStudio (version 2023.06.0), and GraphPad Prism (version 10) were used for analyses.

## Results

### Patient Characteristics

The total study population was 287 patients, with a median age of 55 (IQR 48–63) years and a female to male ratio of 2.19:1. Table 1 provides the baseline characteristics of these study participants, stratified by favorable and unfavorable neurological outcomes. Of these, 176 patients (61.3%) showed a good neurological outcome with an mRS of 0–2. The data showed significant clinical differences between patients with aSAH with good and poor outcome regarding age, Glasgow Coma Scale (GCS) score, the Hunt and Hess score, and CT characteristics on admission. Secondary infarctions were observed in 98 patients (34.1%).

**Table 1:** Baseline characteristics of study population and stratification regarding favorable (mRS 0–2) and poor neurological outcome (mRS 3–6)

	Total study population (*N* = 287)	Favorable outcome (*n* = 176)	Unfavorable outcome (*n* = 111)	*p* value
Age, years, median (IQR)	55 (48–63)	46 (53–59)	60 (50–70)	**< 0.001**^**a**^
Sex, female	197 (68.6)	119 (67.6)	78 (70.3)	0.637^b^
BMI, median (IQR)	25.0 (22.9–27.7)	24.8 (22.9–27.6)	25.4 (22.7–27.7)	0.867^a^
GCS score at admission, median (IQR)	13 (3–15)	14 (9–15)	3 (3–14)	**< 0.001**^**b**^
Hunt and Hess score	–	–	–	**< 0.001**^**b**^
I	54 (18.8)	44 (25.0)	10 (9.0)	–
II	69 (24.0)	54 (30.7)	15 (13.5)	–
III	59 (20.6)	42 (23.9)	17 (15.3)	–
IV	44 (15.3)	17 (9.7)	27 (24.3)	–
V	61 (21.3)	19 (10.8)	42 (37.8)	–
Modified Fisher classification				**< 0.001**^**b**^
I	10 (3.5)	9 (5.1)	1 (0.9)	–
II	12 (4.2)	5 (2.8)	7 (6.3)	–
III	101 (35.2)	76 (43.2)	25 (22.5)	–
IV	124 (57.1)	86 (48.9)	78 (70.3)	–
Intraparenchymal hemorrhage	106 (36.9)	45 (45.6)	61 (55)	**< 0.001**^**b**^
Intraventricular hemorrhage	175 (61)	91 (51.7)	84 (75.7)	**< 0.001**^**b**^
Localization of aneurysm				0.497^b^
Anterior circulation	243 (84.7)	147 (83.5)	96 (86.5)	–
Posterior circulation	44 (15.3)	29 (16.5)	15 (13.5)	–
Treatment of aneurysm	–	–	–	0.057^b^
Clipping	140 (48.8)	78 (44.3)	62 (55.9)	–
Coiling	147 (51.2)	98 (55.7)	49 (44.1)	–

Unless otherwise indicated, data are *n* (%)

*BMI* body mass index, *GCS* Glasgow Coma Scale, *IQR* interquartile range, *mRS* modified Rankin scale

^a^Mann–Whitney *U*-test for independent samples. ﻿Bold values indicate significance (p<0.05)

^b^*χ*^2^ testing

In the Earlydrain trial, 144 patients of the study group were randomized into the LD group and 143 patients were randomized into the standard of care group. The placement of an additional EVD system was at the discretion of the local caregivers. Effectively, 221 patients received an EVD, and 140 patients received an LD. Considering possible combinations, 98 patients were treated with both an EVD and an LD, 42 patients received an LD without an EVD, and 114 patients received an EVD without an LD. Thirty-three patients were managed without any CSF drainage in the first 8 days after aSAH. Ninety-three (52.8%) of the study participants with favorable neurological outcome were treated with CSF drainage through an LD (including patients with LD only and LD and EVD), and 57 (32.4%) of these individuals were treated with both LD and EVD.

Patients in whom the CSF volume was drained both via EVD and LD (*n* = 98) had a median age of 55 (IQR 48–63) years, and 72.4% of these study participants were female. Of these individuals, 12.2% had an aSAH Hunt and Hess grade of 1, 22.4% had a grade of 2, 21.4% had a grade of 3, 17.3% had a grade of 4, and 26.5% had a grade of 5. The location of the corresponding aneurysms in 76.5% of the study participants was in the anterior circulation. Fifty-four (55.1%) of the aneurysms were treated via coiling. Fifty-seven (58.2%) of the patients treated with EVD and LD showed a favorable neurological outcome, and in 30 (30.6%) study participants, a new secondary infarction occurred.

### Total Amount of CSF Drainage

The mean total amount of CSF drainage in the whole study population within the first 8 days after treatment of the SAH was 1052 ± 659 mL. Table 2 presents the drainage amounts of CSF through EVD and LD, categorized by favorable and unfavorable neurological outcomes. There was no difference in overall total CSF drainage between the outcome groups for the whole study population (CSF_total,favorable_ = 997 ± 683 mL; CSF_total,unfavorable_ = 1,139 ± 613 mL; *p* = 0.084). However, the data revealed significantly higher CSF drainage volumes through EVD in patients with poor outcome (CSF_EVD,favorable_ = 589 ± 699 mL; CSF_EVD,unfavorable_ = 868 ± 632 mL; *p* < 0.001) and significantly higher CSF drainage amounts through LD in study participants with good neurological outcome (CSF_LD,favorable_ = 408 ± 456 mL; CSF_LD,unfavorable_ = 271 ± 378 mL; *p* = 0.02). Supplemental Fig. 1 visually depicts the distributions of the drainage volumes, stratified regarding good and poor neurological outcome, for the study population and patients with EVD and LD as density plots. The CSF drainage of the study population divided between good and poor neurological outcome and the occurrence of secondary infarctions are provided in Supplemental Fig. 2. Patients experiencing poor outcomes demonstrated increased daily CSF drainage during the first 8 days following aneurysm treatment. Trends indicate that high levels of EVD may be associated with poor outcome and that high levels of LD may be associated with good outcome.

**Table 2:** Drainage amount of study population and stratification regarding favorable (mRS 0–2) and unfavorable neurological outcome (mRS 3–6)

	Total study population (*N* = 287)	Favorable outcome (*n* = 176)	Unfavorable outcome (*n* = 111)	*p* value^a^
Day 1				
CSF total	124 ± 100	116 ± 104	136 ± 92	**0.043**
CSF EVD	97 ± 99	87 ± 102	112 ± 93	**0.003**
CSF LD	27 ± 44	28 ± 45	24 ± 43	0.186
Day 2				
CSF total	147 ± 106	139 ± 107	162 ± 103	**0.046**
CSF EVD	100 ± 107	85 ± 107	125 ± 104	**< 0.001**
CSF LD	47 ± 62	54 ± 66	37 ± 54	**0.041**
Days 3–5				
CSF total	427 ± 269	406 ± 281	461 ± 246	0.081
CSF EVD	273 ± 285	229 ± 293	344 ± 258	**< 0.001**
CSF LD	153 ± 190	176 ± 198	117 ± 170	**0.100**
Days 6–8				
CSF total	360 ± 267	336 ± 272	399 ± 257	**0.036**
CSF EVD	230 ± 268	187 ± 268	302 ± 255	**< 0.001**
CSF LD	129 ± 184	149 ± 194	97 ± 162	**0.035**
Total amount (days 1–8)				
CSF total	1052 ± 660	997 ± 683	1139 ± 613	0.084
CSF EVD	697 ± 686	589 ± 699	868 ± 632	**< 0.001**
CSF LD	354 ± 432	408 ± 456	271 ± 378	**0.02**

All data are in mL, mean ± SD

*CSF* cerebrospinal fluid, *EVD* external ventricular drainage, *LD* lumbar drainage, *mRS* modified Rankin scale, *SD* standard deviation

^a^Mann-Whitney *U*-test for independent samples. Bold values indicate significance (p<0.05)

**Fig. 1:**
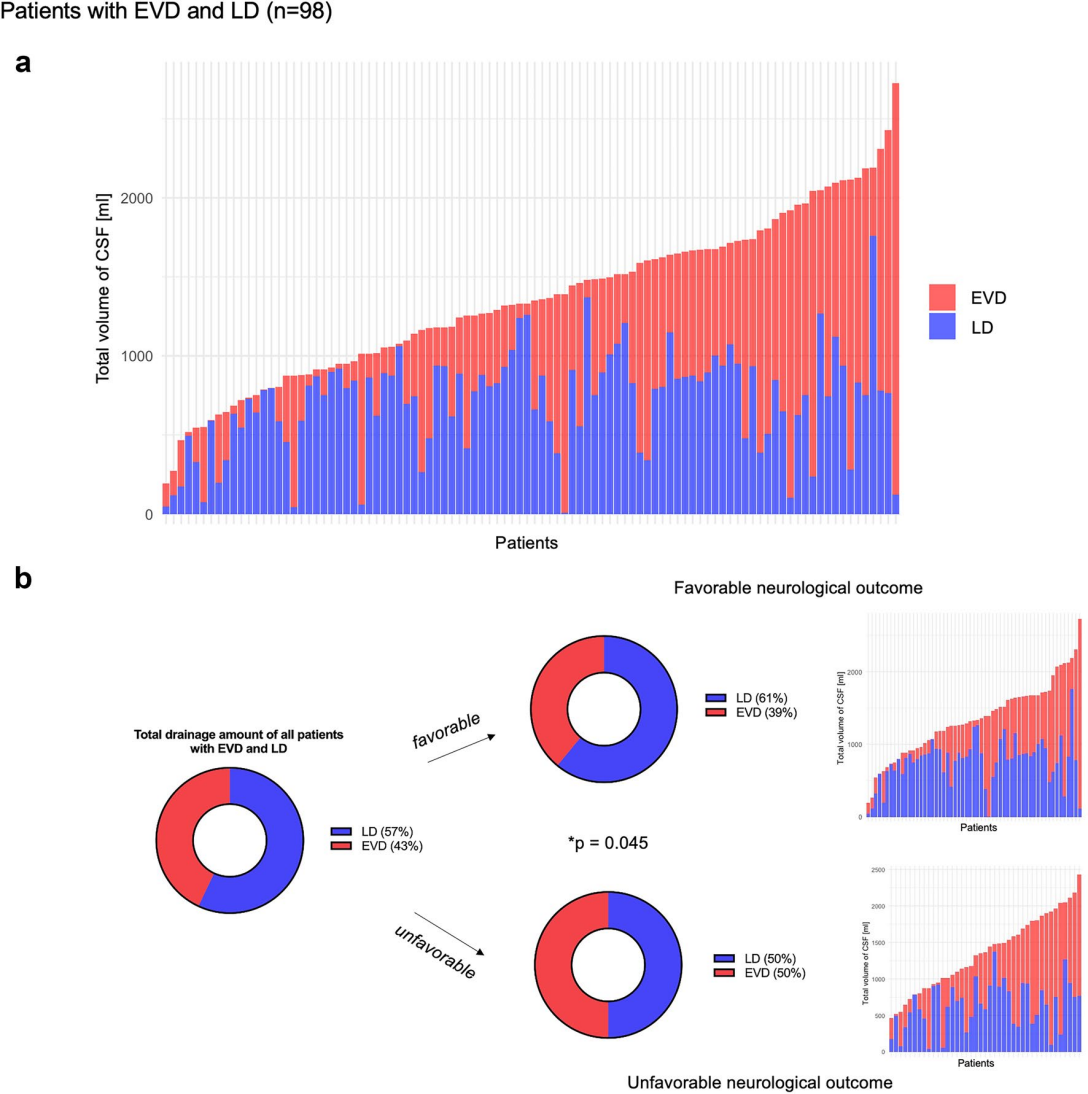
Drainage volumes of patients with EVD and LD (*n* = 98). **a** Drainage volumes of individual patients, sorted in ascending order by the total amount. **b** Mean percentages of CSF drainage through LD and EVD. *CSF* cerebrospinal fluid, *EVD* external ventricular drainage, *LD* lumbar drainage

**Fig. 2:**
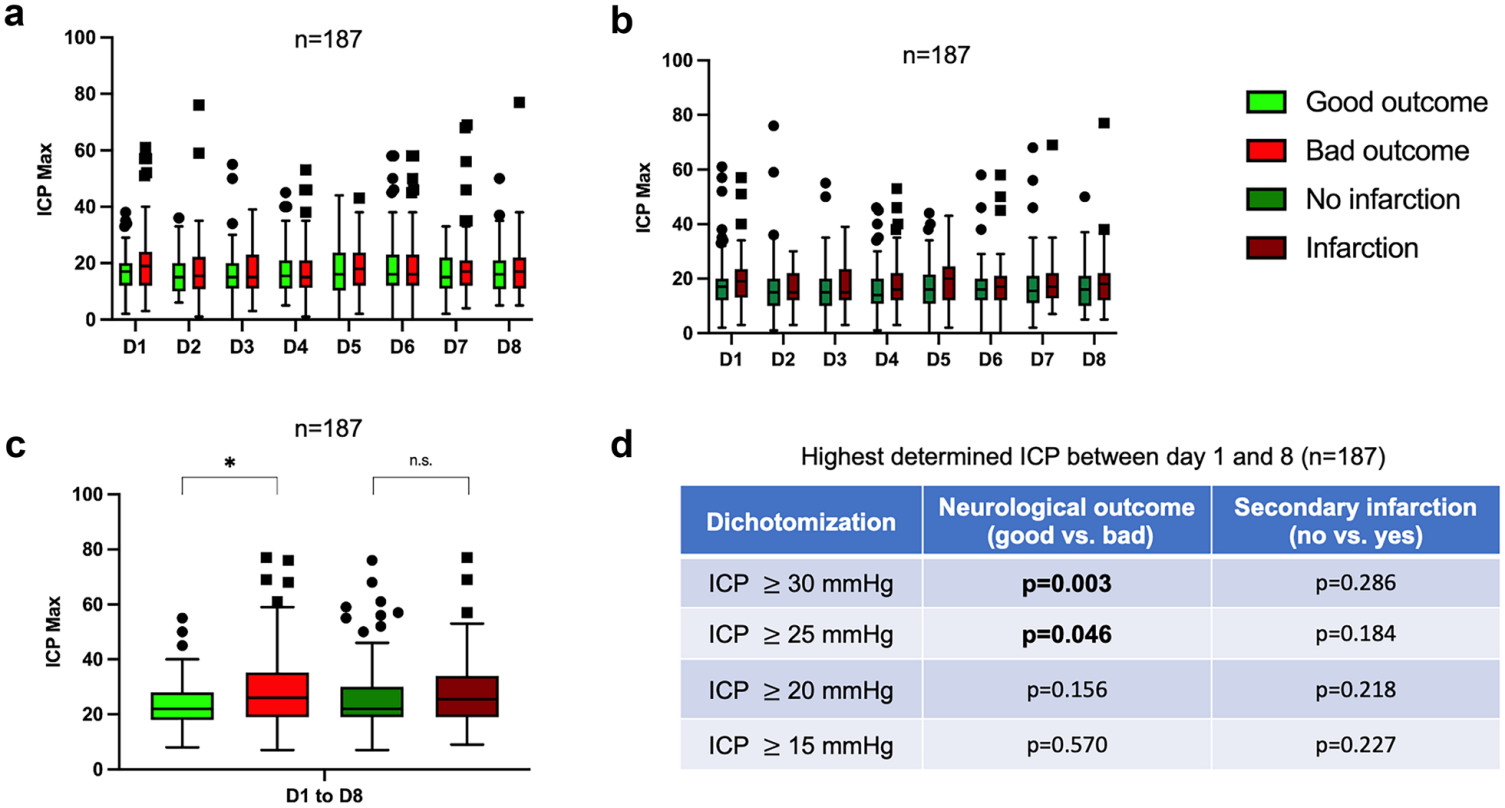
Highest determined ICP of the day of the study population (*n* = 187). The patients are stratified according to the neurological outcome (**a**) (mRS 0–2 = good, mRS 3–6 = bad) and the occurrence of secondary infarctions after treatment of aneurysms (**b**). **c** Highest measured ICP between day 1 and day 8 after the bleeding event. **d** Highest determined ICP between days 1 and 8 of the study population. Dichotomized (ICP ≥ 30 mm Hg vs. < 30 mm Hg, ICP ≥ 25 mm Hg vs. < 25 mm Hg, ICP ≥ 20 mm Hg vs. < 20 mm Hg, and ICP ≥ 15 mm Hg vs. < 15 mm Hg) and stratified according to neurological outcome (mRS 0–2 = good, mRS 3–6 = bad) and the occurrence of secondary infarctions or not. *p < 0.05. *ICP* intracranial pressure, *D* day, *mRS* modified Rankin scale, *n.s.* not significant

In the subsets of patients who received an LD, consisting of patients with only LD and patients with LD and EVD, an average volume of 727 ± 333 mL was drained through the LD in the first 8 days after aneurysm occlusion. These patients demonstrated no difference in the total amount of CSF (CSF_total,favorable_ = 1126 ± 536 mL; CSF_total,unfavorable_ = 1275 ± 541 mL; *p* = 0.128) drained between patients with favorable and unfavorable neurological outcome. However, there was a significant positive effect of LD amount (CSF_LD,favorable_ = 771 ± 334 mL; CSF_LD,unfavorable_ = 640 ± 317 mL; *p* = 0.042) and a significant negative effect of EVD amount on the outcome of this patient group (CSF_EVD,favorable_ = 355 ± 495 mL; CSF_EVD,unfavorable_ = 634 ± 534 mL; *p* < 0.01). Further, for patients receiving both LD and EVD, no significant difference was observed in the total drained CSF volume between those with good and poor neurological outcomes (CSF_total,favorable_ = 1346 ± 513 mL; CSF_total,unfavorable_ = 1,365 ± 512 mL; *p* = 0.891). For these patients, there was no significant difference in the absolute volume drained through the EVD (CSF_EVD,favorable_ = 579 ± 520 mL; CSF_EVD,unfavorable_ = 727 ± 510 mL; *p* = 0.126) and LD (CSF_LD,favorable_ = 767 ± 320 mL; CSF_LD,unfavorable_ = 638 ± 326 mL; *p* = 0.060) concerning the neurological outcome. Nonetheless, the trend observed mirrored those seen in the entire study population. Supplemental Fig. 3 presents the daily drainage amounts for these patients, categorized according to their outcomes.

**Fig. 3:**
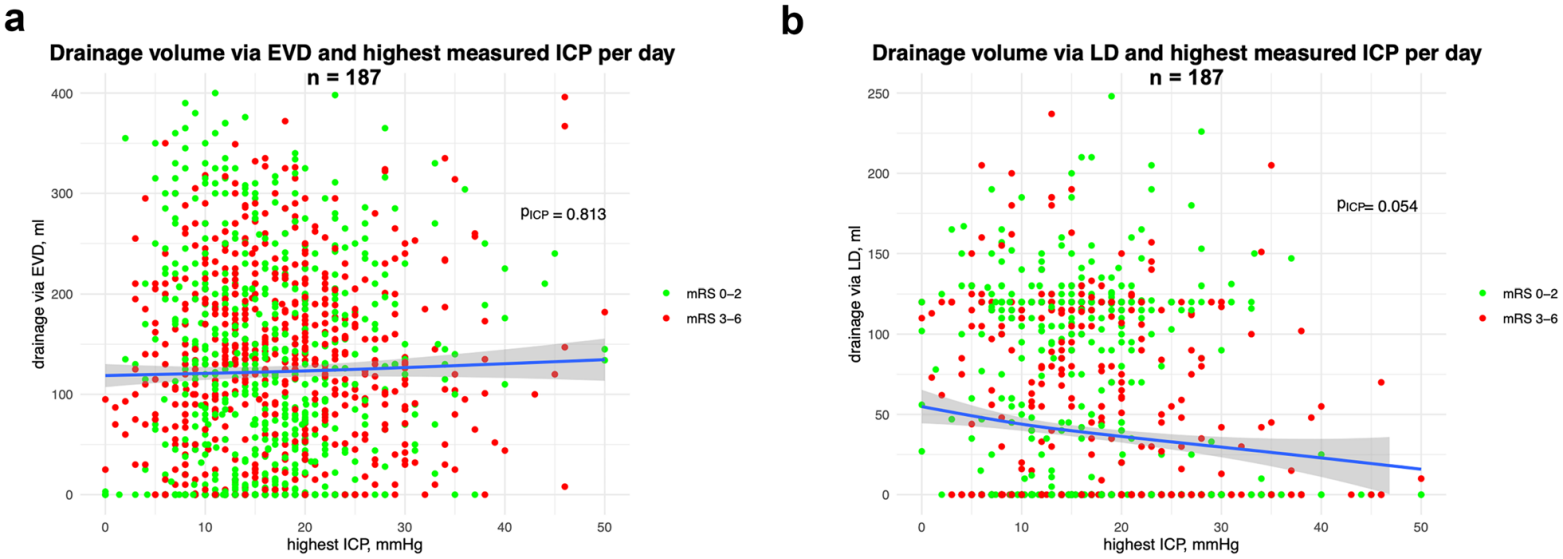
Correlation between highest measured ICP and CSF drainage per day (*n* = 187). **a** Drainage volume via EVD and highest measured ICP per day. **b** Drainage volume via LD and highest measured ICP per day. Significance values are derived from a generalized additive mixed model and adjusted for outcome category. *p* values are derived from a generalized additive model for the correlation of ICP and drainage volume. Adjustment of repeated measurements for outcome analysis was not performed, and accordingly, no *p* value for differences in outcome is reported. *CSF* cerebrospinal fluid, *EVD* external ventricular drainage, *ICP* intracranial pressure, *LD* lumbar drainage

Of all patients in whom the CSF volume was drained through an LD (*n* = 140), 93 (66.4%) individuals showed favorable outcome. Therefore, patients who needed only an LD without an EVD (*n* = 42, favorable neurological outcome: 85.7%) showed better neurological outcome than patients who needed drainage through both an LD and an EVD (*n* = 42, favorable neurological outcome: 58.2%; *p* = 0.002).

### Percentage of CSF Drainage Volume Through LD

In patients in whom CSF was drained both through EVD and LD systems, study participants with a higher percentage of CSF drainage through the LD experienced better neurological outcomes (*p* = 0.045). The mean total CSF drainage volume measured was 1,354 ± 510 mL. The mean drainage volumes via EVD and LD were estimated to be 641 ± 518 mL and 713 ± 327 mL, respectively. Figure 1 summarizes the drainage volumes and percentages of CSF for patients who were treated with EVD and LD. The individual patients were sorted by the amount of total drained CSF. The figure demonstrates that, although a regular drainage of 5 mL/h was targeted, the LD volume was heterogeneous in different patients. Furthermore, the percentage of the LD volume was also significantly enhanced in patients with better neurological outcome (*p* < 0.01) for the whole study population.

### Drainage Amount as an Independent Predictor for Neurological Outcome and Secondary Infarctions

To evaluate potential outcome prediction performances of CSF volumes after aSAH, ROC calculations were performed. Therefore, the total drainage amount did not serve as a predictor after aSAH concerning the neurological outcome (AUC_CSF,total_ 56.1% [95% confidence interval (CI) 49.4–62.7%]). EVD volume predicted unfavorable neurological outcome with an AUC_EVD_ of 64.3% (95% CI 58.0–70.7%) and secondary infarction with an AUC_EVD_ of 60.3% (95% CI 53.6–67.0%). The corresponding ROC graph is provided as Supplemental Fig. 4.

**Fig. 4:**
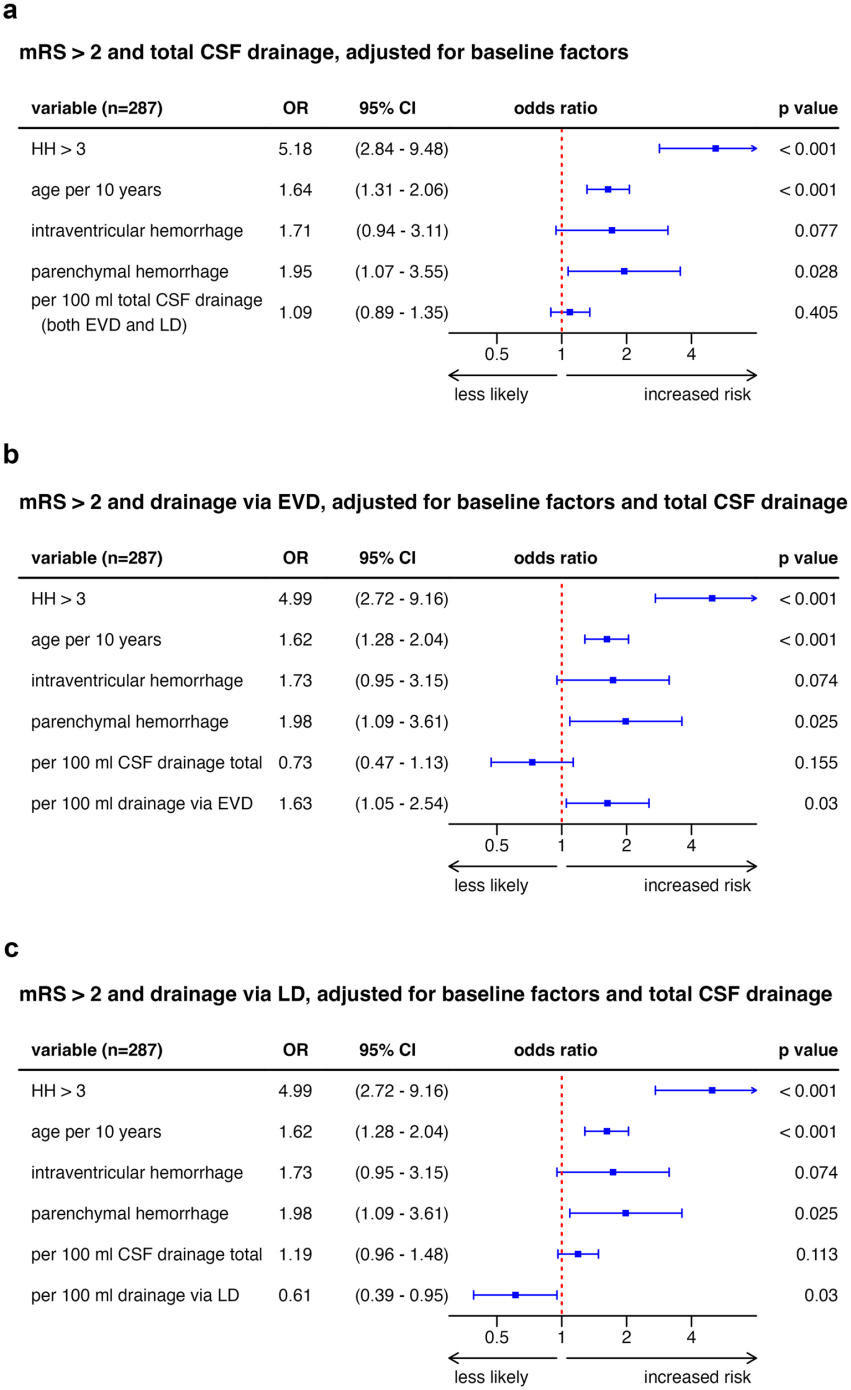
OR plots of the study population (*N* = 287) concerning the neurological outcome of the study participants, with a higher OR indicating worse outcome. Multivariate adjustment for total CSF drainage (**a**), EVD amount (**b**), and LD amount (**c**). Note the similarity of baseline factor ORs in each plot, whereas ORs for LD and EVD amounts point in opposite directions. *CI* confidence interval, *CSF* cerebrospinal fluid, *EVD* external ventricular drainage, *HH* Hunt and Hess grade, *LD* lumbar drainage, *mRS* modified Rankin scale, *OR* odds ratio

### ICP After aSAH

Data on ICP values were available for 187 patients, all of whom were included in the ICP analysis. We observed no significant difference in a routinely performed spot measurement of ICP at 7 a.m. for the patients at the first 8 days after the bleeding event in relation to their neurological outcome (Supplemental Fig. 5). Furthermore, daily maximum ICP measurements showed no differences between patients with favorable and unfavorable outcome (Fig. 2a, b). However, the overall highest ICP value recorded between day 1 and day 8 post bleeding was significantly higher (*p* < 0.001) in patients with unfavorable neurological outcomes. Regarding the occurrence of infarctions, no difference was found in the maximum ICP values (Fig. 2c). When dichotomized according to a threshold of a maximum measured ICP level ≥ 25 mm Hg, patients with higher ICP demonstrated significantly worse neurological outcomes compared with those with lower ICP levels (Fig. 2d). Figure 3 depicts the wide scatter of the highest ICP values and CSF drainage amounts, segmented according to the neurological outcome of the patients. In patients with a higher maximum ICP, no higher treatment amount of EVD was found, whereas the rate of LD was lower.

### Association of Daily Data and Outcome, Adjusted for Baseline Hemorrhage Severity

Individual daily drainage and ICP data and their association with neurological outcomes and the occurrence of infarctions are detailed one-by-one in Table 3. For these analyses, repeated assessments with daily measurements in each patient were considered. If an EVD or LD was not present on a given day, an amount of zero drainage was assumed. The differences concerning the drainage amount remained significant after adjustment for known predictors for the severity of the SAH on admission (Hunt and Hess grade > 3, age, intracerebral bleeding, and intraventricular bleeding).

**Table 3:** One-by-one association of CSF drainage and ICP data with outcome

Variable	*n*	OR_univariate_	95% CI_univariate_	*p*_univariate_	OR_adjusted_	95% CI_adjusted_	*p*_adjusted_
Neurological outcome (mRS 0–2 vs. 3–6)							
Total CSF per 100 mL	287	1.193	0.988–1.44	0.07	1.093	0.887–1.346	0.4
EVD per 100 mL	287	1.409	1.151–1.724	< 0.001	1.268	1.022–1.573	0.03
LD per 100 mL	287	0.637	0.452–0.897	0.01	0.664	0.433–1.02	0.06
ICP at 7 a.m	187	1.039	1.001–1.08	0.05	1.034	0.995–1.075	0.09
Highest ICP	187	1.023	0.999–1.049	0.06	1.022	0.998–1.047	0.08
Secondary infarction							
Total CSF per 100 mL	287	1.073	0.888–1.297	0.46	1.002	0.819–1.227	0.98
EVD per 100 mL	287	1.289	1.058–1.570	0.01	1.203	0.974–1.484	0.09
LD per 100 mL	287	0.594	0.416–0.849	0.004	0.603	0.411–0.883	0.009
ICP at 7 a.m	187	1.048	1.008–1.09	0.02	1.038	0.998–1.08	0.06
Highest ICP	187	1.018	0.996–1.042	0.11	1.102	0.991–1.033	0.27

Adjusted values are corrected for Hunt and Hess grade > 3, age, intracerebral hemorrhage, and intraventricular hemorrhage

*CI* confidence interval, *CSF* cerebrospinal fluid, *EVD* external ventricular drainage, *ICP* intracranial pressure, *LD* lumbar drainage, *mRS* modified Rankin scale, *OR* odds ratio

Multivariable odds ratio plots for drainage volumes in the whole study population with adjustment for total drainage volume are provided in Fig. 4. Visually striking is the similarity of baseline factors in each graph, whereas the odds ratios of LD and EVD amounts point in opposite directions. The total EVD and LD amount combined, again, was not significantly associated with outcome. A multivariate analysis of drainage amounts together with ICP data was not considered meaningful because of the lack of ICP data in about one third of patients.

## Discussion

The principal finding of this study is that in patients with better neurological outcome, higher amounts and percentages of lumbar CSF were drained. This may indicate a potential quantity-dependent protective effect through LD. Further, our data demonstrated that ICP elevations above 25 mm Hg are associated with poor neurological outcome after SAH. ICP elevations and total drainage amount were not significantly related.

The Earlydrain study, a randomized controlled trial, demonstrated that prophylactic use of LDs leads to less infarctions and improved outcome after aSAH, based on the intention-to-treat principle [17]. The present analysis aims to analyze actually recorded ICP data and drainage volumes of this trial regardless of the assigned randomization group, to find additional physiologic explanations on possible mechanisms of action. The results are in line with contemporary work concerning the outcome distribution and the incidence of secondary infarctions [12, 23–26] and The data reveal differences in CSF drainage via EVD and LD within the first 8 days after hemorrhage in patients with varying outcomes. They also highlight disparities in CSF drainage methods among patients with or without secondary infarctions following aSAH.

The daily CSF drainage﻿ volumes observed in the present study align with comparable studies and meta-analyses investigating the CSF drainage amount and regimens after aSAH [10, 27, 28]. A high CSF drainage amount through EVD is associated with worse neurological outcome and more secondary infarctions, whereas patients with better outcome show more amounts of CSF via LD. In Earlydrain, the study protocol aimed for a fixed controlled lumbar CSF drainage of 5 mL/h. It is tempting to speculate that patients requiring more drainage were simply more affected by the initial hemorrhage. Under a fixed CSF LD regimen, this may necessitate more EVD drainage to control ICP, resulting in more CSF drainage through EVD in patients with poor outcome. Direct comparison of ICP data and drainage volumes rejects this assumption. Because the cumulative total volume of CSF in patients with favorable and unfavorable outcome was equivalent in this study, the authors do not consider hemorrhage severity as an underlying cause for EVD drainage but rather attribute the outcome results as a beneficial effect of the LD. In multivariate analysis, we did adjust for initial hemorrhage severity, but findings remained unchanged. The total amount of CSF drainage was not associated with infarction or neurologic outcome before and after adjustment for baseline SAH severity (Table 3). This is also corroborated by the fact that the present data show a higher proportion of lumbar drainage of the total CSF amount in patients who exhibit favorable outcome. The reasons behind the potential quantity-dependent protective effect through LD can only be speculated by the authors. In addition to improved clearance of blood and its degradation products [17, 29], CSF also contains a multitude of immunological components, such as a peak presence of highly inflammatory neutrophil extracellular traps observed on day 7 after aSAH [30]. Increased lumbar drainage may potentially mitigate effects of the innate immune system. However, this remains speculative, and further translational research is necessary and crucial.

Accurate prediction of neurological outcome of patients with aSAH is still difficult. In complex models, for example, based on machine learning approaches, including radiographic, clinical, and laboratory parameters can enhance prediction performances in various clinical settings [23, 31]. Drainage volumes have primarily been used to predict the need for ventriculo-peritoneal shunting following aSAH [32]. The current data show that drainage amount stratified by route serves as a potential parameter for the prediction of neurological outcome and secondary infarctions in patients with aSAH. The Earlydrain study protocol limited lumbar drainage to instances when the gradient between ICP measured by EVD and LD was less than 5 mm Hg. New approaches using oscillation analysis may potentially indicate safe lumbar drainage when the gradient is > 5 mm Hg or when no EVD is present [33]. This may lead to an enhanced proportion of lumbar drainage  and could potentially raise the positive outcome effects in future extended LD regimens. Possible explanations for the better neurological outcome through more lumbar drainage were not part of this investigation; however, one possible explanation may be an accelerated reduction of subarachnoid clots through LD [16]. The outcome predication performance with an AUC of about 65% by analyzing only the EVD drainage of the patients within the first 8 days after bleeding is weak but shows comparable results to outcome predictions solely based on individual parameters as the modified Fisher score or the Barrow Neurological Institute score [23]. Thus, it may be beneficial to incorporate the CSF drainage amount through EVD and LD into complex, multiparametric predictive machine learning models to improve the outcome prediction [34]. However, when used as single marker, these values have limited predictive power.

Exacerbation of ICP is a common complication after aSAH, and sufficient management is crucial for the therapy [10, 11]. The mounting importance of ICP management becomes evident considering the guidelines for therapy after SAH: Older guidelines [20] did not make assertions regarding ICP, whereas more recent guidelines [35, 36] strongly recommend strict ICP management. However, precise target ranges for ICP are not specified. The present data indicate that patients who have even once exceeded an ICP ≥ 25 mm Hg are associated with unfavorable neurological outcome. Rationale may be that ICP spikes trigger spreading depolarizations [37]. These recurrent, intensive, widespread depolarization waves originate from metabolically compromised brain regions, extend deep into healthy tissue, and are considered as precursors of impending infarction [38]. Interestingly, our data indicate that ICP levels showed a wide scatter and no clear relationship to EVD volumes. Higher LD rates tended to decrease measured ICP. It is debated whether CSF drainage should be performed continuously or intermittently [10, 11]. To detect and prevent pressure peaks ≥ 25 mm Hg, intermittent drainage when only an EVD and no parenchymal sensor is placed for ICP measurement may be useful. Predominantly closed drains facilitate ICP monitoring with intermittent CSF drainage on demand and exhibit less device malfunctioning and infections [39]. Furthermore, they may be helpful to assess safety in the Earlydrain concept by calculation of the gradient between EVD and LD.

This study has several limitations. Only the drainage amount and ICP in the first 8 days were recorded. Because secondary brain injury often occurs later, it is possible that the analyzed values do not represent these later events. An important limitation of the present data is that only single snapshot values of ICP were recorded. Consequently, no analyzing of ICP waves and duration of exceeding certain values was possible. Within this study, controlled lumbar drainage was not performed when the ICP was ≥ 20 mm Hg, even if the difference to the EVD was < 5 mm Hg. Thus, it cannot be fully ruled out that less lumbar drainage simply indicated frequently higher ICP. However, the ICP analysis did not provide any evidence for this, and an analysis adjusted for severity of the diseases provided no evidence that this played a role in the present analysis. Furthermore, the protocol for the placement of EVDs and invasive ICP probes was left to the discretion of the treating centers. The lack of information regarding the placement and usage of invasive ICP probes represents a limitation of the present study. A primary limitation to be noted is that patients requiring only an LD may generally exhibit a more benign disease course because otherwise an additional EVD would have been placed. Assumptions on ICP values could be weakened when measurement was performed intermittently. For the correlation analysis between ICP and drainage volume, an amount of zero drainage was assumed if an EVD or LD was not present on a given day. The implications of this assumption are not definitively assessable and should therefore be regarded as a limitation concerning the analysis. In *χ*^2^ testing in few cases, table cell frequencies were less than five. In these cases, Fisher’s exact test was used.

For patients with traumatic brain injury, analysis of continuous ICP monitoring shows that time spans of exceeding pressure thresholds are outcome relevant [40]. These effects could not be investigated and depicted within the context of this study and may also explain why only trends, but no outcome-relevant significances, could be detected when analyzing a snapshot ICP value at 7 a.m. The current data show no significant association between elevated ICP and secondary infarctions, despite the suggested triggering of spreading depolarizations by ICP crises [37]. ICP was consistently higher in patients treated without LD [17]. A notable limitation regarding the ICP monitoring is the absence of information regarding consciousness and sedation status. Especially in conscious patients, relevant confounders could potentially impact the ICP. Secondary infarctions were characterized as infarctions observed on postprocedural CT scans following aneurysm occlusion. This is different from the definition of delayed cerebral ischemia by Vergouwen et al. [41]. This may be regarded as a limitation, but with a multitude of definitions for delayed cerebral ischemia being available in the literature, in our opinion, it may add clarity in distinguishing clinical signs for ischemia and radiological infarction.

Delayed cerebral ischemia typically occurs commonly 4–14 days after the bleeding event and thus later than the granular treatment data recorded in Earlydrain [12, 17, 42]. Besides vasospasms, several vascular and neural pathomechanisms have been described as potential causes of ischemia [12, 43, 44]. The data in the Earlydrain trial [17] showed that the maximum levels of transcranial doppler (TCD) within the first 8 days after the bleeding event did not differ between patients with additional lumbar CSF drainage and standard of care; however, TCD values were higher in patients with secondary infarctions, indicating that vasospasms may play a role. Further localization of secondary infarctions diagnosed in the last cerebral imaging was not recorded. Therefore, no analysis was possible regarding the territory of the infarction. Finally, we did not record information on the presence of hydrocephalus, which may have triggered implementing and the amount of drainage via an EVD. Also, the way of EVD, whether open or closed, was at the discretion of individual centers and was not recorded.

## Conclusions

ICP elevations above 25 mm Hg are associated with poor neurological outcome after SAH. The overall drainage volume amount after aSAH does not differ concerning the outcome of the study participants. Patients with better outcome show higher levels and percentages of CSF drainage through a lumbar system, indicating a potential quantity-dependent protective effect. Possible future studies may investigate different LD regimens with higher amounts of removal. Based on the findings of this study, we hypothesize that maximizing the volume drained through LD and routinely performed measurement of ICP to stringently prevent ICP peaks exceeding 25 mm Hg may be a strategy to improve patient outcomes after aSAH.

## Supplementary Information

Below is the link to the electronic supplementary material.

Supplementary file1 (pdf 1531 KB)
