# Supplementary material for: Analysis of Cerebral Spinal Fluid Drainage and Intracranial Pressure Peaks in Patients with Subarachnoid Hemorrhage
Source: Neurocrit Care. 2024 Apr 15;41(2):619–31. doi: 10.1007/s12028-024-01981-9 (PMC11377663; doi:10.1007/s12028-024-01981-9)
Supplement: Supplementary file 1 — Supplementary file1 (pdf 1531 KB) [file 12028_2024_1981_MOESM1_ESM.pdf]

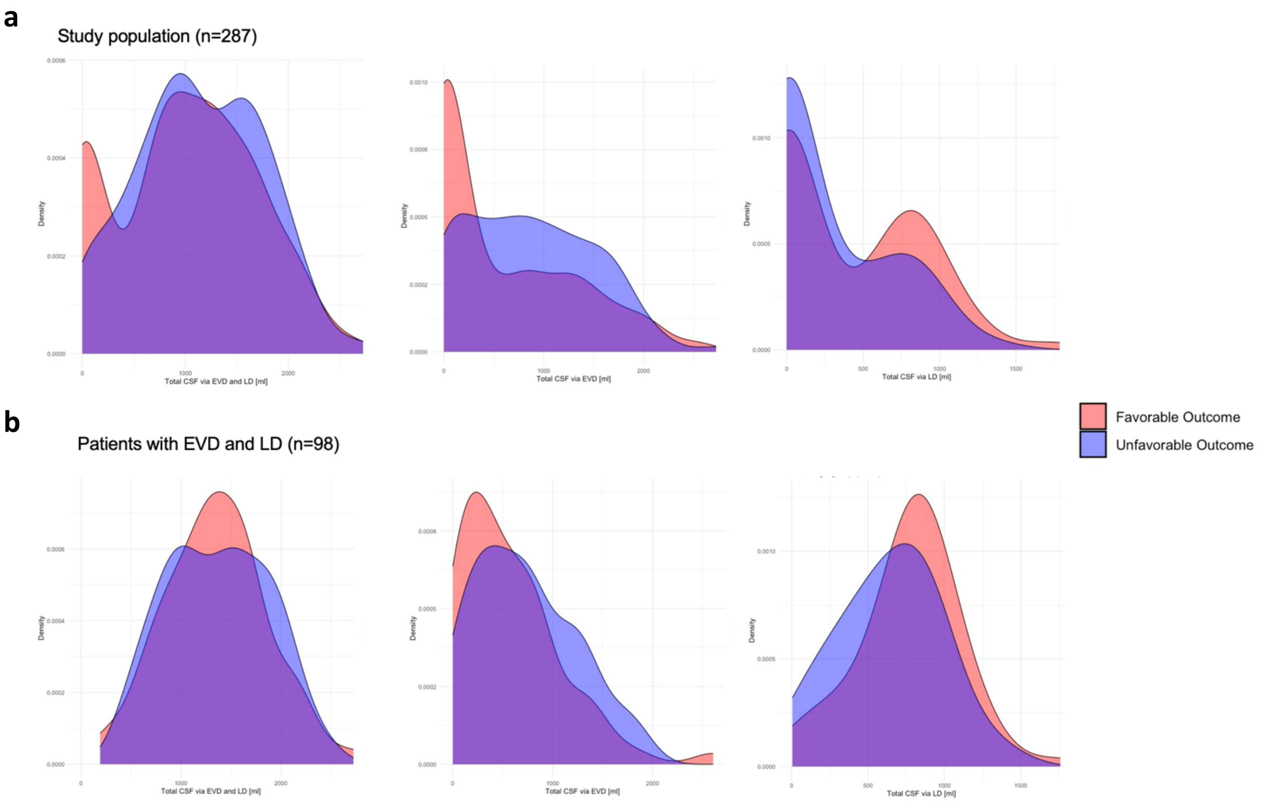

Supplemental Figure 1. Density plot of the drainage volumes of the whole study population (n=287, a) and patients with both EVD and LD (n=98, b). CSF: Cerebral spinal fluid, EVD: External ventricular drainage, LD: Lumbar drainage

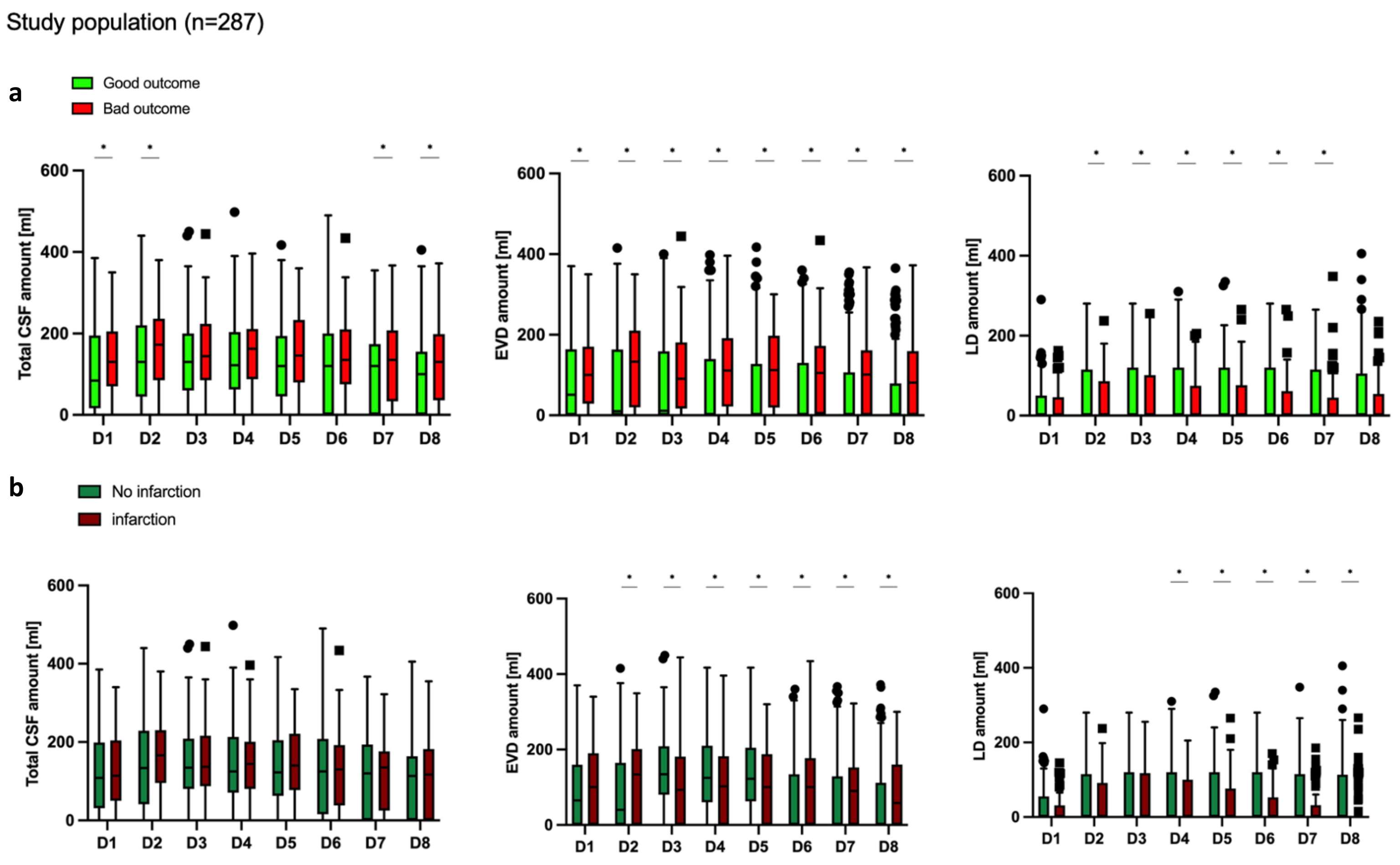

Supplemental Figure 2. Daily amount of CSF Drainage in the whole study population (n=287). a) Stratified according to the neurological outcome (mRS 0-2 = favorable, mRS 3-6 = unfavorable). b) Stratified according to the occurrence of secondary infarctions after treatment of aneurysms. *p<0.05, CSF: Cerebral spinal fluid, EVD: External ventricular drainage, LD: Lumbar drainage, D: day

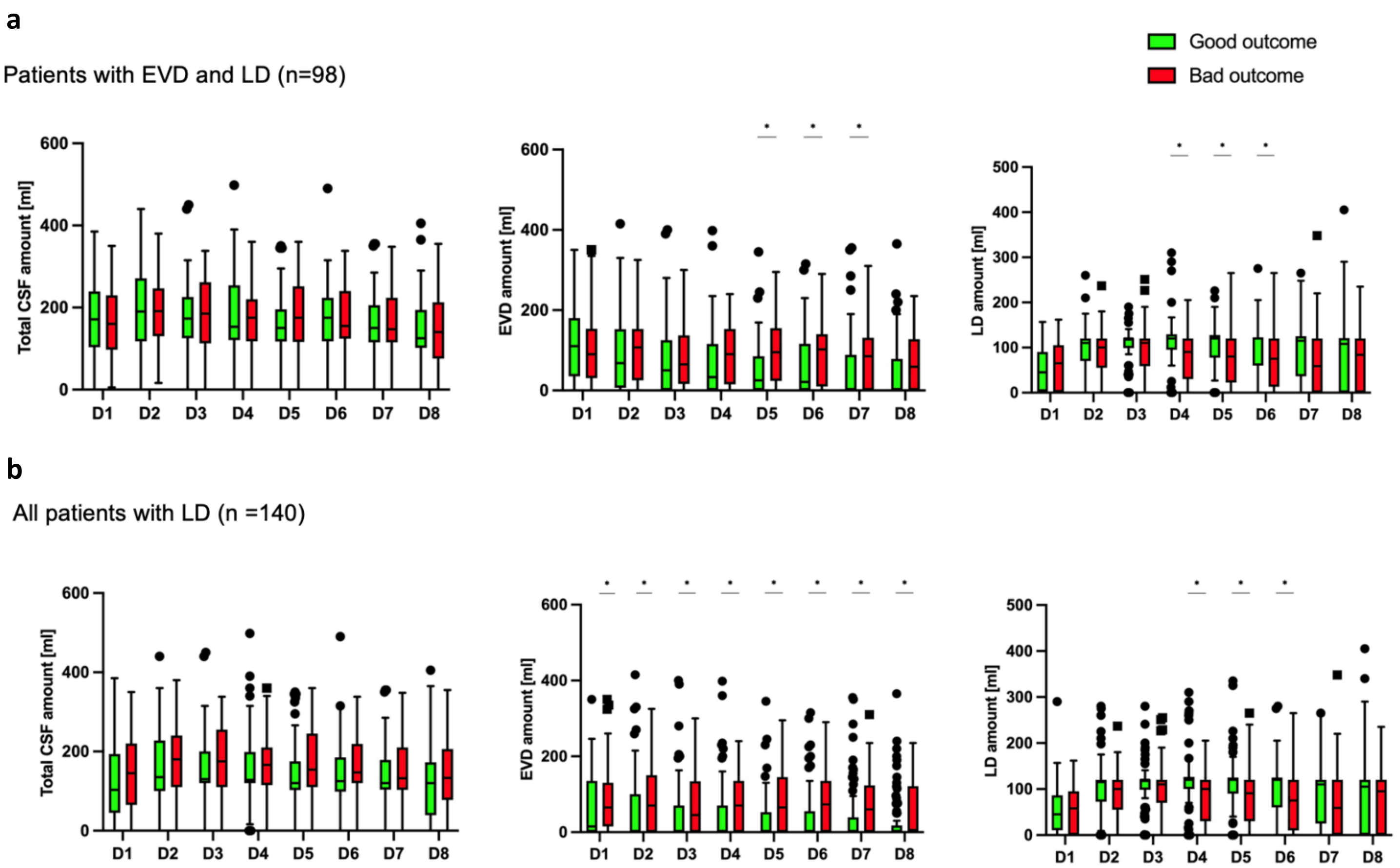

Supplemental Figure 3. Daily amount of CSF Drainage of the patients with both an EVD and LD (n=98, a) and in all patients treated with LD, regardless whether an EVD is present (n=140, b). Stratified according to the neurological outcome (mRS 0-2 = good, mRS 3-6 = unfavorable). In the "all patients with LD" group EVD amount was counted as zero when no EVD was in place. *$p<0.05$, CSF: Cerebral spinal fluid, EVD: External ventricular drainage, LD: Lumbar drainage, D: day

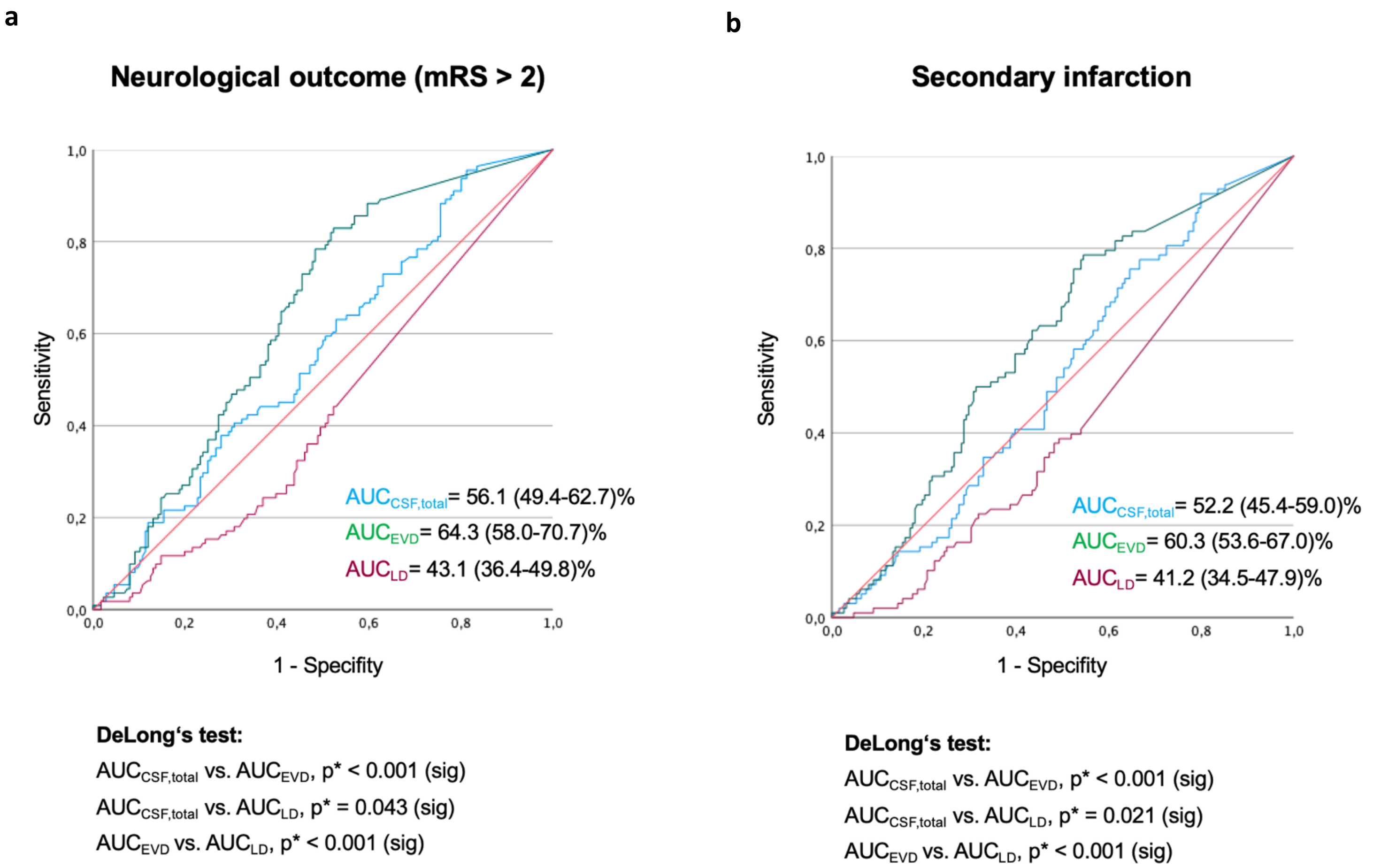

Supplemental Figure 4. a) Prediction of unfavorable outcome (mRS > 2) and b) secondary infarction based on CSF amount of the whole study population (n=287). AUC: Area under the curve, CSF: Cerebral spinal fluid, EVD: External ventricular drainage, LD: Lumbar drainage

a b

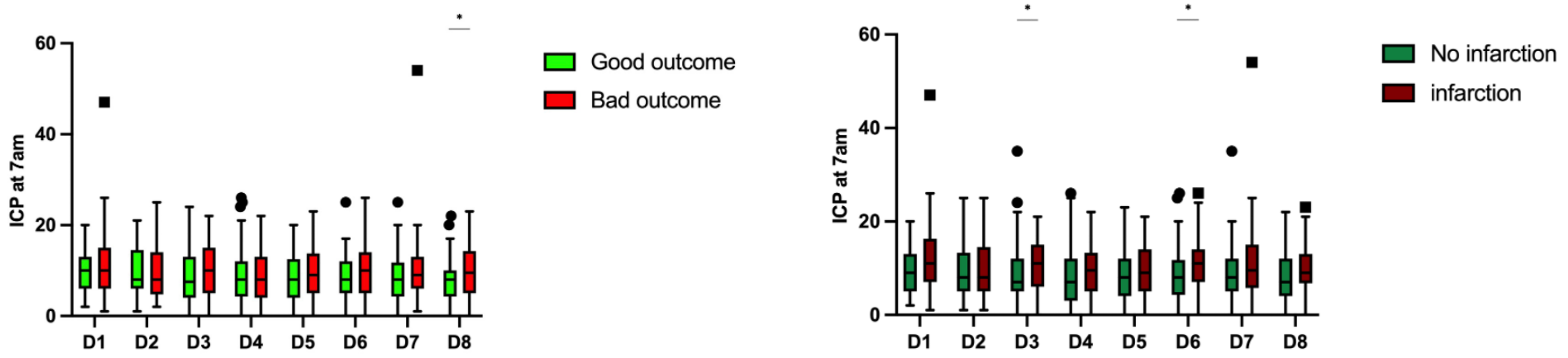

Supplemental Figure 5. Daily determined ICP of the patients at 7am (n=187). The patients are stratified according to a) the neurological outcome (mRS 0-2 = good, mRS 3-6 = bad) and b) occurrence of secondary infarctions after treatment of aneurysms.

*p<0.05, ICP: Intracranial pressure, D: day

Supplemental Table 1. Drainage amount of study population and stratification regarding occurrence of new infarction or not

	Total study population (n= 287)	No infarction (n = 189)	infarction (n = 98)	p value[T]
**Day 1**				
CSF total	124 ± 100	121 ± 102	130 ± 98	0.451
CSF EVD	97 ± 99	90 ± 98	110 ± 100	0.068
CSF LD	27 ± 44	31 ± 47	20 ± 37	0.063
**Day 2**				
CSF total	147 ± 106	141 ± 109	161 ± 98	0.105
CSF EVD	100 ± 107	89 ± 107	121 ± 104	**0.105**
CSF LD	47 ± 62	52 ± 64	39 ± 57	0.076
**Day 3 to 5**				
CSF total	427 ± 269	421 ± 278	438 ± 253	0.735
CSF EVD	273 ± 285	247 ± 291	323 ± 268	**0.008**
CSF LD	153 ± 190	173 ± 196	115 ± 171	**0.014**
**Day 6 to 8**				
CSF total	360 ± 267	357 ± 277	364 ± 248	0.636
CSF EVD	230 ± 268	203 ± 274	283 ± 250	**0.001**
CSF LD	129 ± 184	153 ± 200	81 ± 135	**< 0.001**
**Total amount (Day 1 to 8)**				
CSF total	1052 ± 659	1032 ± 688	1089 ± 602	0.539
CSF EVD	694 ± 687	626 ± 700	827 ± 647	**0.04**
CSF LD	257 ± 432	406 ± 457	262 ± 363	**0.09**

Abbreviations: CSF: Cerebral spinal fluid, EVD: External ventricular drainage, LD: Lumbar drainage, SD: Standard deviation. All data in ml, mean ± SD. [T]Mann-Whitney-U-testing for independent samples. Bold values indicate significance ($p < 0.05$)
